# Effects of Combination Treatment on Renal Endothelial Function in Type 2 Diabetes Mellitus

**DOI:** 10.1016/j.ekir.2025.07.023

**Published:** 2025-07-23

**Authors:** Merve Günes-Altan, Agnes Bosch, Kristina Striepe, Mario Schiffer, Roland E. Schmieder, Dennis Kannenkeril

**Affiliations:** 1 Department of Nephrology and Hypertension, University Hospital Erlangen, Friedrich-Alexander-University Erlangen-Nürnberg, Erlangen, Germany; 2 Department of Cardiology, University Hospital Erlangen, Friedrich-Alexander-University Erlangen-Nürnberg, Erlangen, Germany

**Keywords:** renal endothelial function, renal hemodynamics, SGLT-2 inhibitors, type 2 diabetes

## Abstract

**Introduction:**

Recently, we demonstrated that a combination therapy with empagliflozin and linagliptin (E+L) in patients with type 2 diabetes mellitus (T2DM) induce changes in renal hemodynamics. The purpose of the present study was to analyze the influence of nitric oxide (NO) activity of the renal vasculature on the described changes of the renal hemodynamic profile.

**Methods:**

Patients with T2DM were randomized to receive either E+L: (*n* = 34) or metformin and insulin glargine (M+I: *n* = 31), for 3 months. Renal hemodynamics were assessed using the constant-infusion input-clearance technique with p-aminohippuric acid for renal plasma flow (RPF) and inulin for glomerular filtration rate (GFR) at baseline and after treatment. Intraglomerular hemodynamics were calculated according to the model established by Gomez. The NO activity in the renal circulation was assessed by analyzing change in RPF in response to i.v. administrated NG-monomethyl-l-arginine (L-NMMA), an NO inhibitor.

**Results:**

After 3 months of treatment, changes in renal hemodynamic parameters were compared with baseline in both groups without any change in renal NO activity. In patients with E+L treatment, we observed a correlation between change in NO activity of the renal vasculature and change in RPF (*r* = −0.665, *P* < 0.001) after 3 months of treatment. Similar correlations with change in renal vascular resistance (RVR) (*r* = 0.439, *P* = 0.003) and resistance of the efferent postglomerular (R_E_) arterioles (*r* = 0.513, *P* = 0.002) were observed. No such relationships with change in renal NO activity were observed in the M+I group after 3 months of treatment.

**Conclusion:**

Renal NO activity emerged as a determinant of the renal hemodynamic response in the combination therapy of E+L, but not in the combination therapy of M+I. Our study provides evidence that the treatment effect of sodium-glucose cotransporter-2 (SGLT-2) inhibitors may be contributed at least partly by the renal NO activity in patients with T2DM.

## Graphical abstract

**Figure:**
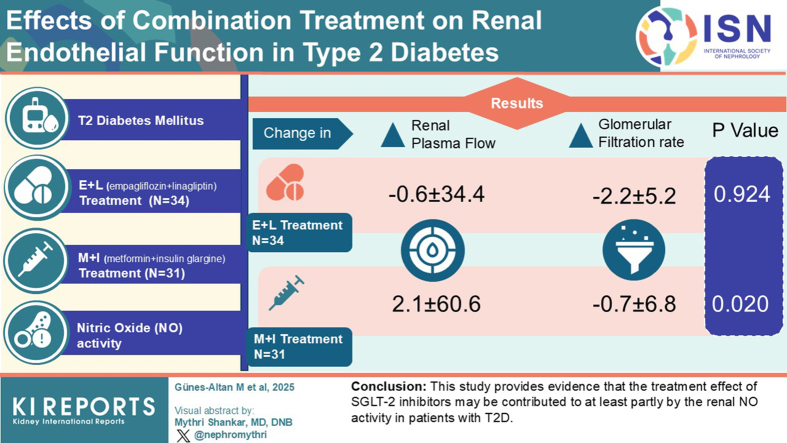

SGLT-2 inhibitors have shown cardiovascular and renal benefits beyond glycemic effects.^1^, ^2^, ^3^, ^4^, ^5^, ^6^, ^7^ The renoprotective effects of SGLT-2 inhibitors attenuate the progression of diabetic nephropathy and reduce risk of adverse renal outcomes in patients with T2DM.^1^^,^^2^^,^^4^, ^5^, ^6^, ^7^ However, the exact mechanisms underlying these renoprotective effects of SGLT-2 inhibitors remain elusive.

The endothelium-derived NO plays an important role in the renal vasculature and is widely used as a marker of renal endothelial function.^8^, ^9^, ^10^, ^11^, ^12^ It is generated by endothelial NO synthase and leads to vasodilatation in the renal vasculature. In the early stages of diabetic nephropathy, an increase in NO leads to glomerular hyperfiltration; whereas in later stages of chronic glomerular disease, NO availability is reduced due to increased oxidative stress.^12^, ^13^, ^14^ However, data on the contribution of NO to glomerular hemodynamics in humans are scarce due to methodological difficulties. Systemic inhibiton of endothelial NO synthase by L-NMMA, which leads to a reduction in RPF, has been used in several human studies to assess the contribution of NO to renal perfusion.^8^^,^^12^^,^^15^, ^16^, ^17^, ^18^, ^19^

Two independent research groups found that renal hemodynamic changes induced by SGLT-2 inhibitors are characterized by a postglomerular vasodilatation.^20^^,^^21^ To elucidate the role of NO activity in mediating any renal hemodynamic changes, we analysed whether the observed treatment effects of the combination therapy, E+L on renal and intraglomerular hemodynamics^20^ are related to NO activity in the renal vasculature in patients with T2DM. In our own previous work, we demonstrated that linagliptin did not significantly alter GFR, RPF, or RVR, so we postulated that the findings of the present *post hoc* analysis can be attributed to empagliflozin.^19^

## Methods

### Study Design

This single-center, *post hoc* analysis includes patients with T2DM who either received E+L or M+I (control group) for 3 months between April 2016 and November 2018. The study was conducted at the Clincal Research Center, Department of Nephrology and Hypertension, University Hospital Erlangen, Germany. The trial was registered at Clinical trials.gov (NCT02752113). The results of the primary study concerning the renal hemodynamics and vascular effects of these antidiabetic combination strategies have been reported previously.^20^^,^^22^ Written informed consent was obtained from all patients before study inclusion. The study protocol was approved by the Local Ethics Committee (University Erlangen-Nuremberg) and the study was conducted according to the Declaration of Helsinki. Metformin medication was stable for at least 3 months in all patients (850 or 1000 mg orally twice daily). Following the stable metformin phase, patients in the E+L group received 10 mg empagliflozin and 5 mg linagliptin orally once daily and metformin was discontinued. Empagliflozin was uptitrated to 25 mg if patients had a fasting blood glucose ≥ 110 mg/dl. Patients in the M+I group received 2 to 4 units of insulin subcutaneously once daily, which was adjusted every third day by adding 2 units if fasting blood glucose was not ≤ 125 mg/dl. The patients in the M+I group continued on metformin in addition to receiving insulin. The M+I group served as a control group in this study. The rationale for selecting these combination therapies was to compare the renal hemodynamic effects of a contemporary standard treatment for T2DM (SGLT-2 inhibitor combined with a dipeptidyl peptidase-4 inhibitor) with those of a more traditional regimen consisting of insulin and metformin.

### Study Cohort

Patients with T2DM aged 27 to 75 years participated in the study. Patients had to have a glycated hemoglobin level ≥ 6.5% if they were on antidiabetic monotherapy and ≥ 6.0% for those receiving dual antidiabetic therapy. The main exclusion criteria were use of insulin, glitazones, dipeptidyl peptidase-4 inhibitors, or SGLT-2 inhibitors within 2 months prior to randomization. Patients with congestive heart failure New York Heart Association III or IV, glycated hemoglobin > 10.5%, fasting plasma glucose > 240 mg/dl, urinary albumin-to-creatinine ratio > 300 mg/g, estimated GFR < 60 ml/min per 1.73 m^2^, body mass index > 40 kg/m^2^ were excluded. A negative pregnancy test was mandatory for female patients before and during the study period.

### Assessments

Renal hemodynamics were assessed using the constant-infusion input-clearance technique with p-aminohippuric acid (Daiichi Sankyo, Tokyo, Japan) for RPF and inulin (Inutest; Fresenius, Linz, Austria) for GFR at baseline and 3 months after treatment initiation.^8^^,^^23^^,^^24^ Due to withdrawal of inulin from the market during the study, GFR was measured only in a subgroup of patients (E+L: *n* = 34; M+I: *n* = 31). NO activity in the renal circulation was assessed by analysing change in RPF in response to i.v. administrated L-NMMA (an NO inhibitor; Clinalfa AG, Läufelfingen, Switzerland) in the subgroup only. The procedure began with a bolus injection of inulin and p-aminohippuric acid, administered over a period of 15 minutes, followed by a continuous infusion for 105 minutes. After steady state was achieved, 2 blood samples were drawn 5 minutes apart to determine RPF and GFR. Subsequently, a bolus infusion of L-NMMA (3 mg/kg body weight over 5 minuntes) was administered i.v. followed by a constant infusion (1.25 mg/kg body weight over 25 minutes), while a constant infusion of inulin and p-aminohippuric acid was continued. Thus, the total dose of L-NMMA was 4.25 mg/kg body weight.^11^^,^^25^ After the steady state was achieved, blood samples were collected again. Intraglomerular hemodynamics were calculated according to the model established by Gomez.^26^ NO activity in the renal circulation was assessed by analyzing the change in RPF in response to i.v. administrated NO inhibitor.

### Statistical Analysis

Statistical analysis was performed using SPSS Statistics 28.0 (IBM, Armonk, NY) and data were expressed as mean ± SD in text and tables. A 2-sided *P*-value < 0.05 was considered statistically significant. Paired *t* test was applied for the comparison of the end of 3 months treatment phase versus baseline within each treatment group. We performed unpaired *t* test to determine the statistical significance of the differences between the E+L and M+I groups. Bivariate correlation analyses for the relationship between the change in NO activity and renal and intraglomerular hemodynamics were assessed by performing Pearson’s test.

## Results

### Clinical Characteristics and Baseline Renal Hemodynamic Parameters

The clinical characteristics and baseline renal hemodynamic parameters of the 2 study groups are given in Tables 1 and 2. The mean age in both groups was 59 years with a 77% male predominance. Baseline RPF was 597.3 ± 108.3 ml/min in patients with E+L treatment and 611.8 ± 128.0 ml/min in patients with M+I treatment (*P* = 0.623, Table 2). There were no differences in demographic data or baseline renal hemodynamic parameters between the 2 treatment groups.

**Table 1:** Baseline characteristics

Parameters	E+L (*n* = 34)	M+I (*n*=31)	*P*-value
Demographic data			
Age (yrs)	59.4 ± 8.4	59.9 ± 9.7	0.807
Gender (m/f)	27/7	23/8	0.618
Weight (kg)	90.6 ± 15.1	94.9 ± 18.4	0.306
BMI (kg/m^2^)	30.2 ± 3.6	31.6 ± 3.8	0.138
Laboratory values			
Fasting plasma glucose (mg/dl)	158.4 ± 27.9	159.8 ± 26.0	0.836
HbA1c (%)	7.7 ± 0.6	7.8 ± 0.7	0.665
Total cholesterol (mg/dl)	197.1 ± 38.4	198.3 ± 31.1	0.896
LDL cholesterol (mg/dl)	129.0 ± 30.1	130.2 ± 24.3	0.858
HDL cholesterol (mg/dl)	47.4 ± 10.5	45.2 ± 11.0	0.410
Office BP			
Systolic BP (mm Hg)	135.1 ± 10.8	134.9 ± 10.3	0.922
Diastolic BP (mm Hg)	82.4 ± 8.2	81.9 ± 10.6	0.835
Heart rate (bpm)	74.5 ± 11.8	72.1 ± 11.6	0.406
Ambulatory BP			
24-h systolic BP (mm Hg)	130.2 ± 10.7	131.3 ± 9.8	0.654
24-h diastolic BP (mm Hg)	81.5 ± 7.1	81.8 ± 8.0	0.884
24-h heart rate (bpm)	76.1 ± 9.4	75.3 ± 10.9	0.751

BMI, body mass index; BP, blood pressure; bpm, beats per minute; E+L, empagliflozin+linagliptin; HbA1c, glycated hemoglobin; HDL, high-density lipoprotein; LDL, low-density lipoprotein; M+I, metformin+insulin glargine.

Data are presented as mean ± SD.

**Table 2:** Baseline renal hemodynamic parameters E+L versus M+I

Parameters	*n*	E+L	*n*	M+I	*P*-value
RPF (ml/min)	34	597.3 ± 108.3	31	611.8 ± 128.0	0.623
RPF (ml/min/BSA)	34	500.0 ± 121.6	31	501.3 ± 138.4	0.881
GFR (ml/min)	34	126.5 ± 13.0	31	126.5 ± 14.8	0.991
GFR (ml/min/BSA)	34	105.5 ± 17.9	31	103.3 ± 19.1	0.730
FF (%)	34	21.6 ± 3.1	31	21.2 ± 2.8	0.536
RVR (mm Hg)	34	93.6 ± 20.1	31	93.3 ± 19.3	0.948
R_A_ (dyn∗s/cm^5^)	34	2547.4 ± 880.2	31	2578.9 ± 798.4	0.952
R_E_ (dyn∗s/cm^5^)	34	2360.9 ± 407.1	31	2315.8 ± 350.7	0.636
P_glom_ (mm Hg)	34	61.6 ± 3.3	31	61.7 ± 3.1	0.900
R_E_/R_A_	34	1.0 ± 0.4	31	1.0 ± 0.3	0.746

BSA, body surface area; E+L, empagliflozin+linagliptin; FF, filtration fraction; GFR (ml/min), measured GFR; GFR (ml/min per 1.73 m^2^), BSA-indexed GFR; GFR, glomerular filtration rate; M+I, metformin+insulin glargine; P_glom_, intraglomerular pressure; R_A_, resistance of afferent arteriole; R_E_, resistance of efferent arteriole; RVR, renal vascular resistance.

Data are presented as mean ± SD.

### Effect of Treatment in Renal Hemodynamic Parameters

The detailed effects of E+L and M+I on renal hemodynamic parameters have been reported previously.^20^ Briefly, in the subgroup of patients that could be examined with inulin clearance to determine GFR, treatment with E+L caused a decline in GFR (*P* = 0.003), RVR (*P* = 0.001), and R_E_ (*P* = 0.001) without any change in RPF (*P* = 0.536) or resistance of afferent arterioles (R_A_) (*P* = 0.116). Notably, renal hemodynamic changes were also observed in the M+I treatment group. In patients with M+I treatment, we observed a decline in RPF (*P* < 0.001), GFR (*P* = 0.001), R_E_/R_A_ quotient (*P* = 0.004). RVR (*P* = 0.001), R_A_ (*P* = 0.006) increased with M+I.^20^ After administration of L-NMMA, all renal hemodynamic parameters, including RPF, declined significantly in both groups (all *P* < 0.001).

### Effect of E+L or M+I on Renal NO Activity

Administration of L-NMMA caused a decline in RPF in all study phases in both treatment groups. In the E+L treatment group, RPF decreased by −48.6 ± 25.6 ml/min after application of L-NMMA before treatment and by −49.1 ± 30.1 ml/min 3 months after treatment initiation (Table 3). Comparing the decline in RPF after application of L-NMMA at baseline and after E+L treatment, we observed no significant difference (−0.6 ± 34.4 ml/min, *P* = 0.924). We also observed no significant differences in the effect of L-NMMA before and after treatment on R_A_, intraglomerular pressure, and R_E_/R_A_ in the E+L group (Table 3). In contrast, comparison of the effects of L-NMMA on GFR, FF, and RE after treatment with E+L showed a significantly lower change in GFR, FF, and RE after treatment (Table 3). In contrast, there were no differences in the effects of L-NMMA between baseline and after treatment in any parameters in the M+I group (Table 3). To compare the treatment effect, the changes in renal hemodynamic parameters following the administration of L-NMMA, at baseline and after 12 weeks of treatment, were compared between the groups (Table 3: change in E+L vs. change in M+I). No significant difference was observed between the groups.

**Table 3:** Effect of E+L or M+I versus baseline on renal NO activity

Parameter	E+L group (*n* = 34)				M+I group (*n* = 31)				Change E+L vs. M+I *P*-value
	Baseline	12 wks	Change	*P*-value	Baseline	12 wks	Change	*P*-value	
Δ RPF (ml/min)	−48.6 ± 25.6	−49.1 ± 30.1	−0.6 ± 34.4	0.924	−43.4 ± 51.4	−41.3 ± 30.8	2.1 ± 60.6	0.849	0.826
Δ GFR (ml/min)	9.5 ± 4.9	7.4 ± 4.9	−2.2 ± 5.2	0.020	9.8 ± 7.2	9.0 ± 4.1	−0.7 ± 6.8	0.551	0.338
Δ FF (%)	3.7 ± 1.1	3.3 ± 1.0	−0.4 ± 1.1	0.033	3.5 ± 1.6	3.5 ± 1.5	−0.03 ± 1.8	0.923	0.279
Δ R_A_ (dyn∗s/cm^5^)	1012.1 ± 573.2	866.4 ± 451.9	−145.7 ± 543.6	0.070	995.0 ± 708.1	1019.3 ± 648.0	24.3 ± 658.4	0.838	0.263
Δ R_E_ (dyn∗s/cm^5^)	478.8 ± 158.0	389.1 ± 116.9	−89.7 ± 151.6	0.002	454.0 ± 208.0	454.2 ± 228.9	0.2 ± 238.4	0.996	0.072
Δ P_glom_ (mm Hg)	2.3 ± 1.4	1.8 ± 1.2	−0.4 ± 1.6	0.105	2.4 ± 1.6	2.2 ± 1.2	−0.2 ± 1.6	0.462	0.622
Δ R_E_/R_A_	−0.1 ± 0.2	−0.2 ± 0.2	−0.04 ± 0.2	0.905	−0.1 ± 0.1	−0.1 ± 0.2	0.01 ± 0.2	0.665	0.324

E+L, empagliflozin+linagliptin; FF, filtration fraction; GFR, glomerular filtration rate; L-NMMA, NG-monomethyl-l-arginine; M+I, metformin+insulin glargine; P_glom_, intraglomerular pressure; R_A_, resistance of afferent arteriole; R_E_, resistance of efferent arteriole; RPF, renal plasma flow; RVR, renal vascular resistance; Δ, change with administration of L-NMMA (an NO inhibitor).

Data are presented as mean ± standard deviation.

### Relationship Between Renal Hemodynamics and Renal NO Activity With Treatment (E+L or M+I)

**Figure 1:**
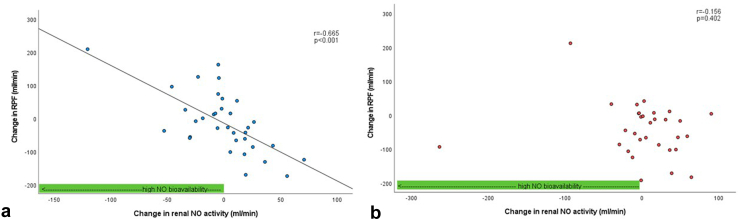
Correlation between change in RPF and change in renal NO activity after 3 months of treatment. (a) Treatment with empagliflozin+linagliptin (E+L). (b) Treatment with metformin+insulin. MNO, nitric oxide; RPF, renal plasma flow.

**Figure 2:**
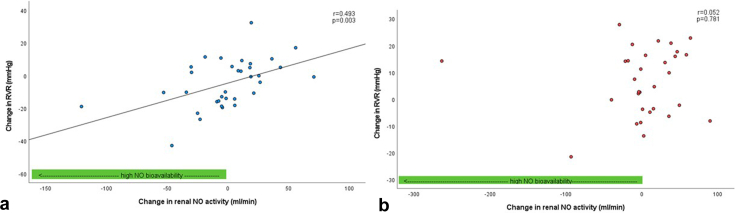
Correlation between change in RVR and change in renal NO after 3 months of treatment. (a) Treatment with empagliflozin+linagliptin (E+L). The correlation remains significant after removal of the outlier (*r* = −0.559, *P* < 0.001). (b) Treatment with metformin+insulin. NO, nitric oxide; RVR, renal vessel resistance.

**Figure 3:**
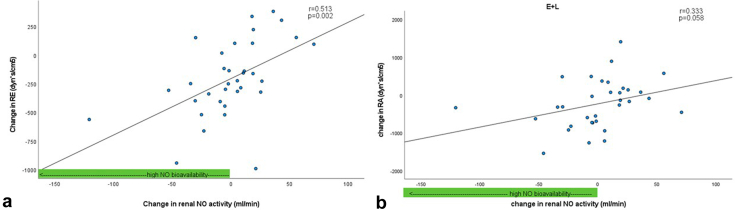
Correlation between change in intraglomerular hemodynamics and change in renal NO activity after 3 months of empagliflozin+linagliptin treatment. (a) Correlation between change in resistance of R_E_ and change in renal NO activity. (b) Correlation between change in resistance of R_A_ and change in renal NO activity. NO, nitric oxide; R_A_, renal afferent arterioles; R_E_, renal efferent arterioles.

We performed correlation analyses between renal NO activity (as assessed by the change of RPF to administration of L-NMMA) before and after treatment and the change in different renal hemodynamic parameters after 3 months of therapy. We observed a significant correlation between change in renal NO activity (difference of the change in RPF after administration of LNMMA pretreatment and change in RPF after administration of LNMMA after treatment) and the change in RPF in the E+L group after treatment (*r* = −0.665, *P* < 0.001, Figure 1a) but not in the M+I group (*r* = −0.156, *P* = 0.402, Figure 1b). Similarly, we observed a correlation between the change in renal NO activity and the change in RVR and R_E_ to treatment only in the E+L group (*r* = 0.493, *P* = 0.003, Figure 2a and *r* = 0.513, *P* = 0.002, Figure 3a) and not in the M+I group (*r* = 0.052, *P* = 0.781, Figure 2b and *r* = 0.221, *P* = 0.233). We also observed a correlation between change in renal NO activity and the change in filtration fraction in the E+L group (*r* = 0.585, *P* < 0.001), but not in the M+I group (*r* = 0.270, *P* = 0.141). Although not statistically significant, we observed a trend for the correlation of the change in R_A_ after therapy and change in renal NO activity in the E+L group (*r* = 0.333, *P* = 0.058, Figure 3b) but not in the M+I group (*r* = −0.076, *P* = 0.686). We did not find a correlation between change in GFR and renal NO activity in either of the groups (E+L: *r* = −0.135, *P* = 0.446; M+I: *r* = 0.068, *P* = 0.716).

## Discussion

Findings related to the renal hemodynamic effects of these antidiabetic combination strategies (E+L vs. M+I) from the primary study have already been reported.^20^ Briefly, M+I therapy was associated with reduced renal perfusion and elevated RVR. In contrast, E+I treatment maintained renal perfusion and lowered RVR. Furthermore, M+I primarily increased R_A_, whereas E+I mainly reduced R_E_. The main finding of our present *post hoc* analysis is that the observed effects of combination treatment E+L on renal perfusion and on preglomerular and postglomerular resistances are at least partly determined by renal NO activity. Such a relationship was not observed after the combination treatment with M+I. Our data further suggest that the renoprotective effects of SGLT-2 inhibitors may be mediated to a certain degree by renal NO activity.

In our study, we investigated renal NO activity by analyzing the change in RPF after L-NMMA infusion, which is an established method to analyze renal endothelial function of the renal vasculature *in vivo* in humans.^8^^,^^13^^,^^15^, ^16^, ^17^, ^18^^,^^27^^,^^28^ In accordance with other research groups, we used the change of RPF in response to L-NMMA administration as a biomarker of renal NO activity, and not the change in GFR, although we observed a significant difference in the change in GFR after L-NMMA administration before and after E+L treatment.^16^^,^^18^^,^^28^

In the present study, renal NO activity (as assessed by applying L-NMMA infusion) caused a similar decrease in RPF before and after treatment with E+L. This lack of difference in the decrease of RPF after administration of L-NMMA before and after E+L could be due to heterogenous and individually variable responses to treatment with E+L between the study patients, as indicated by the fact that the response of RPF to L-NMMA infusion before and after treatment did not correlate with each other (*r* = 0.243, *P* = 0.166). Schlaich *et al.* previously showed in 310 subjects with normal renal function that there is a wide range of renal NO activity in the study population.^12^ However, despite the fact that we did not observe any change in basal NO activity after treatment with E+L compared with pretreatment, we noted a close relationship between the change in renal NO activity after therapy and change in renal perfusion and intrarenal resistances. In our study, we not only observed a correlation between the change in renal NO activity after therapy and change in RPF due to therapy, but also between change in renal NO activity and the change in RVR and filtration fraction after treatment with E+L. An increase in renal NO activity was related to a decrease in RVR, in particular, of the postglomerular arterioles at the postglomerular efferent site.

In accordance with our interpretation, Delles *et al.*^16^ previously investigated the effects of treatments with enalapril, eprosartan, and a combination of both on renal hemodynamics. As in our study, renal NO activity was measured via systemic application of L-NMMA and by analyzing the change in RPF.^16^ Similar to our results, Delles *et al.*^16^ did not observe any change in renal NO activity, but observed a correlation between renal NO activity and increase in RPF after treatment with eprosartan or combination treatment, and concluded that the angiotensin receptor blocker, eprosartan increased renal NO activity.

Previous experimental trials in animals have shown that renal NO has predominant effects on the afferent arteriole.^29^^,^^30^ In our study, we only observed a significant relationship between decrease in R_E_ and increase in NO activity. About the R_A_, we only observed a trend but not a significant correlation. Schlaich *et al.* previously showed that renal NO activity has effects on the afferent and efferent glomerular resistance arterioles, with a predominant effect of renal NO activity on the afferent preglomerular site.^12^ Similarly, Delles *et al.*^8^ observed that the afferent arteriole appears to be the predominant site of NO activity rather than the efferent arteriole. Previously, it has been demonstrated that L-NMMA administration increases both the resistances of afferent and efferent arterioles in isolated perfused kidney.^31^ However, we observed a close relationship between renal NO activity and postglomerular resistance, specifically after the combination therapy with E+L, a different situation from those in above quoted studies. Furthermore, we observed a decrease in RPF and an increase in GFR and filtration fraction after administration of L-NMMA, that can only be explained by a greater increase of the R_E_ than of the R_A_. In summary, we suggest that after combination treatment E+L, renal NO activity is exerting its vasodilating effect predominantly on the postglomerular efferent arterioles.

In comparison to the E+L treatment group, although we observed significant changes in RPF, GFR, RVR, R_A_ and R_E_/R_A_ after 3 months of treatment with M+I, we did not find any correlation between the change in renal NO activity and the change in renal hemodynamic parameters. This discrepancy between the 2 treatment arms underlines the specificity of the observed relationship between the renal NO activity and the renal hemodynamics in patients with E+L treatment. The interpretation of the hemodynamic changes in the M+I group should consider potential vascular effects of insulin. Although insulin has been described to exert both vasodilatory and vasoconstrictive effects^32^^,^^33^ depending on timing of administration and concentration, our previous data^20^ showed an increase in afferent resistance under insulin therapy.

Considering that linagliptin has not been shown to impact renal NO activity according to our previous study, we suggest that the SGLT-2 inhibitor, empagliflozin affects predominantly renal NO activity.^19^

### Limitations

Our study has several limitations. We analyzed patients receiving a combination treatment with E+L; thus, it is not possible to definitively attribute the treatment effects E+L treatment on renal NO activity only to empagliflozin. However, Ott *et al.*^19^ previously showed that renal NO activity as assessed by applying L-NMMA infusion (as done in the current study) does not improve from baseline after treatment with linagliptin. Thus, we assume that the correlation of increase in renal NO activity with increase in RPF, decrease in RVR, and decrease in RE in this study are primarily attributed to empagliflozin and not to linagliptin. The relationship between the changes in renal hemodynamics induced by E+L treatment and NO activity remains unclear, with no indication as to causality or potential mediating mechanisms between these factors. Furthermore, our study does not provide data on renal risk over time. Therefore, we cannot draw any conclusions about a potential correlation or mediating role of renal NO activity on renal risk. Another limitation of our study is that we did not assess daily protein and salt intake, which may influence the renal hemodynamics.^34^^,^^35^ Our results are only valid for patients with early stage of T2DM, having estimated GFR > 60 ml/min per 1.73 m^2^ and cannot be attributed to other forms of diabetes or patients with chronic kidney disease.

## Conclusion

Our data indicate that the renal protective effects of SGLT-2 inhibitors may be mediated by changes in renal NO activity, and that renal NO activity not only determines renal but also intraglomerular hemodynamics. In conclusion, our study provides evidence that the treatment effect of SGLT-2 inhibitors may be contributed at least partly by the renal NO activity in patients with T2DM.

## Disclosure

No funding was received for this study. RES reports grants to the institution to and as speaker and adviser to Boehringer Ingelheim. DES reports receiving grants to the institution from Boehringer Ingelheim. All the other authors declared no competing interests.

## Data Availability Statement

The datasets used and/or analyzed during the current study are available from the corresponding author on reasonable request.
